# Epidemics and Ambulances

**Published:** 1889-12-28

**Authors:** 


### The Hospital

A Weekly Journal of

Science, Medicine, Nursing, and Philanthropy.

*For Hospitals, Asylums, Medical Practitioners, Students, Nurses, Families, Parishes, Congregations, and the Charitable Public.*

Vol. VII. No. 170.] [December 28, 1889.

### Epidemics and Ambulances.

Infectious diseases are generally and in their own nature more capable of control and suppression than those of a non-infectious character, and yet the public always feels more afraid of epidemics than of all the other common diseases taken together. An infectious disease may be likened to a fire. So long as the fire is small and confined to a grate, or even when it is large, if it be kept within easily manageable limits, it answers the purpose for which it has been kindled, and does no harm to anybody. It may be surrounded on all sides by materials of the most inflammable kind, such as gunpowder, paraffin, and the like, but if retained under complete control it can by no possibility be injurious. The parallelism between fire and infectious diseases is as complete as it can well be, if modern bacillary science is in any sense practically true. Just as we cannot gather "grapes of thorns nor figs of thistles," so can we not gather scarlet fever, or measles, or smallpox, or any other infectious disease, except from other human beings in whom its germs are developing or have already developed. In another sense it is equally true that typhoid fever and diphtheria cannot be acquired except from the taking into the system of poison germs of such a kind as will produce typhoid or diphtheria and nothing else. In other words, if all persons could be kept from contact with sources of infection they might live for a thousand years in the ordinary atmosphere and circumstances of the world, so far as danger from infectious disease is concerned.

Now, let every non-medical mind consider for a moment what this means to the inhabitants of large centres of population. It means that if they have intelligence enough, and sufficient promptitude of resource, they need never again be at the mercy of a general epidemic of any of the common infectious diseases. The nightmare of dread which always weighs down the anxious parents of large families of children might be entirely and for ever lifted away if those in responsible positions had sufficient knowledge, resources, and energy to cope at once with any threatened outbreak of zymotic disease. The mere knowledge of the possibility of such a state of sanitary perfection gives hope to the most pessimistic, and renews the energy of the feeblest.

It is our happiness, as the French say, to be able to announce a very important departure by the Metropolitan Asylums Board, in the direction of increasing the means of public safety in this regard. The Board, as is well known, has under its control a very efficient system of horse ambulance; and, up to the present time, this has always been at the service of persons requiring to be removed to any of the infirmaries or asylums under the Board's management —that is to say, it has been used for the benefit of the poorest classes of the people, and for their benefit exclusively. At the last meeting of the Board, held on the 30th November, it was decided that in future the "managers do allow their carriages to be made use of for the conveyance of persons suffering from 'dangerous infectious diseases' to places other than the Board's hospitals." The average charge for such removal is to be five shillings. In cases of need, the committee have power to remit even this nominal sum. It has been also resolved to authorise the committee to "convey persons, under exceptional circumstances, to or from places outside the metropolitan area, and to make an extra charge for the same."

This new departure of the Metropolitan Asylums Board demands the immediate consideration of the public from two opposite points of view. The Board are public servants, and it is for those whom they serve to consider whether or not the change shows evidence of zeal and capacity. The question, indeed, needs no discussion. It is seen at once that the Board have done exactly what the highest measure of good sense and a clear appreciation of the public needs would have dictated. From this point of view, its action is entirely satisfactory, and we congratulate both the members and the public on the initiation of a spirited, judicious, and eminently useful policy.

But there is another aspect of the case. Now that these great advantages are placed at the disposal of the metropolis and the surrounding districts, what will the people do with them? Not long ago London adopted the Compulsory Notification of Infectious Diseases Act. The necessary complement of that Act is the means of putting it into practice. By the action of the Metropolitan Asylums Board those means are now placed within reach, not only of every vestry and other public body, but of every householder and lodger within the metropolitan area. Theoretically, therefore, we are prepared not only to deal with epidemics in the promptest and most complete way, but we are as completely prepared to prevent the occurrence of them. The question is, Will epidemics be prevented in future? And that question must be answered by asking another, Will the whole of the public avail themselves of the means now placed at their disposal for promptly isolating cases of infectious disease, and so of preventing the spread of the contagion?

It is satisfactory to know that within the past ten or twelve years a great change has come over public opinion with respect to such questions as compulsory notification and the isolation of the sick. Parents, even among the poorest classes, recognise that when scarlet fever or other similar disease breaks out in the house, the very kindest thing to be done to the sufferer himself is to secure his removal to a well-equipped fever hospital. Whilst from the point of view of other members of the family his retention at home is a terrible danger, to those who are responsible it is little less than a crime. But there is an undoubted urgency that the public should be both better informed and better instructed, not only as to the necessity for the immediate isolation of every sporadic case of infectious disease, but also as to the means available for carrying out the work. "The price of liberty" is said to be "eternal vigilance"; and similarly the price of public health and public safety is eternal instructedness and unceasing watchfulness both on the part of the people themselves and of those who are entrusted with authority and power. By the people especially is it incumbent that the duty of constant watchfulness should be cheerfully accepted, because no zeal on the part of officials, and no system, however perfect, can keep London or any other large city healthy and safe without the spontaneous and loyal co-operation of every well-disposed inhabitant.

The newspapers have an important function to perform in this connection; and it must be admitted that in the past they have performed it with a degree of intelligence and a persistent, though kindly, zeal which could hardly have been anticipated beforehand. Articles on sanitary questions are not lively "copy"; and yet there is hardly a newspaper in London that does not keep its readers well informed on questions of public health, and also well up to date in the newest scientific developments bearing on the subject. For this purely gratuitous service the public cannot easily be too grateful. It is much to be desired that the new departure of the Metropolitan Asylums Board be properly valued by the daily and weekly press; and that from time to time, as opportunity may serve, the public be both reminded and admonished as to its duties. It is by such mutual and reciprocal help as this that vast and concentrated populations ameliorate the sternness of the common lot, and secure that gradual advancement towards a higher and wholesomer civilisation which history and science have taught us to expect.

